# Single-Center Experience with Venus-P Self-expanding Pulmonary Valve: Insights on Valve Sizing and Procedural Techniques

**DOI:** 10.1007/s00246-025-03841-5

**Published:** 2025-04-02

**Authors:** Sok-Leng Kang, J. D. R. Thomson, Phuoc Duong, James R. Bentham

**Affiliations:** 1 Department of Paediatric Cardiology, Leeds Teaching Hospitals Trust, Leeds, UK; 2 Department of Paediatric Cardiology, John Hopkins University Hospital, Baltimore, USA; 3 Department of Paediatric Cardiology, Alder Hey Children’s Hospital, Liverpool, UK

**Keywords:** Transcatheter, Pulmonary valve, Congenital heart disease, Tetralogy of Fallot

## Abstract

We share a single-center experience of transcatheter pulmonary valve replacement (PVR) with the Venus-P valve, discussing procedural insights, considerations for valve sizing in relation to valve expansion and geometry, and associated outcomes. Retrospective review of 42 consecutive cases from January 2017 to March 2024. The median age and weight of the cohort were 39.6 (27.3, 58.6) years and 69.8 (59.8, 85.3) kg and the Venus-P valve was successfully implanted in 98%. Severe adverse events included valve migration requiring surgical explant and PVR (*n* = 1), sustained ventricular arrhythmia (*n* = 1), and endocarditis (*n* = 1). At mean follow-up of 20.3 ± 19.6 months, there was no deterioration in valve performance and statistically significant improvement in NYHA class, right ventricular volumes, biventricular stroke volume, and LV ejection fraction. Cinefluoroscopic measurements of the deployed valves showed highest expansion and lowest eccentricity at valve coaptation level and greater frame deformation at the inflow compared to outflow flare. Supra-annular (*n* = 9) implant showed a higher expansion of the straight valve section compared to transannular (*n* = 32) deployment, related to lesser degree of valve oversizing and tissue characteristics at implant site. Transcatheter PVR with the Venus-P valve can be safely performed in a broad range of anatomies with clinical benefit at medium-term follow-up. Valve sizing focused on adequate deformation of inflow and outflow flares at implant location with minimal constrain at the valve portion and supra-annular positioning, may result in a higher degree of valve expansion and circularity. Ongoing follow-up of this cohort will provide important insights into valve longevity and long-term outcomes.

**Supplementary Information:**

The online version contains supplementary material available at 10.1007/s00246-025-03841-5.

## Introduction

In contemporary congenital heart disease practice worldwide, children and adults with native or post-surgical dysfunctional right ventricular outflow tract (RVOT) represent a growing population. Transcatheter pulmonary valve replacement (PVR) is an important treatment option in the lifetime management strategy of these patients as a less invasive alternative to surgical valve replacement. The balloon-expandable Melody™ (Medtronic, Minnesota, USA) and Edwards SAPIEN valves (Edwards Lifesciences) have proven to be safe and effective for implantation in dysfunctional conduits and bioprosthetic valves, but neither is ideally suited or feasible for chronically regurgitant and expansile native nor patched outflow tracts encountered in many patients requiring PVR.

For these patients, several self-expanding technologies including the Venus-P valve (Venus Medtech, Hangzhou, China) and Harmony valve (Medtronic, Minnesota, USA) have shown promising early result [1, 2]. These prostheses share in common an ‘hourglass’-shaped nitinol stent frame, designed to provide stability and conformability within large diameter native outflow tracts. Compared to the cobalt–chromium frames of balloon-expandable valves, the nitinol frame of self-expanding valves has lower radial strength and final prosthesis configuration is highly dependent on the degree of valve oversizing, elasticity of surrounding tissue or patch material and presence of calcification. While adequate deformation of the inflow and outflow flares are required for fixation, excessive or eccentric deformation at the level of valve coaptation may result in leaflet pinwheeling and restricted motion. The impaired leaflet kinematics increases valve strain and is associated with clinical and subclinical valve thrombosis [3–8]. Thus, decision-making on valve sizing and positioning, as well as anticipation of potential challenges associated with anatomically diverse and dynamic RVOT is important and has implications on hemodynamic outcomes and valve durability [9].

In clinical practice, non-standardization of pre-procedure imaging and heterogeneity in RVOT geometry challenges the development of a specific sizing algorithm for the Venus-P valve. Several centers have reported empirical use of the annulus or balloon waist as the major reference, applying valve oversizing in the range of 2–5 mm larger than the balloon waist [1, 10, 11]. Given that the annulus is frequently noncircular, this approach results in differing degrees of oversizing when measured in different planes and may result in unnecessary under-expansion or asymmetric expansion at the valve level. In this context, we sought to share our experience with the use of Venus-P valve, including approach to valve selection, positioning and technical modification in relation to hemodynamic and clinical outcomes in a series of patients with native dysfunctional RVOT.

## Methods

### Study Design and Population

This is a retrospective, single-center cohort study of all adult patients who underwent transcatheter PVR with Venus-P Valve implantation from January 2017 to March 2024. In our institution, decision for PVR is made based on patient symptoms and MRI criteria outlined in the ESC and AHA guidelines within a joint adult and pediatric congenital multidisciplinary team meeting. Our unit preference is for percutaneous PVR where technically possible, based on magnetic resonance imaging (MRI) or computed tomography (CT) assessment of RVOT morphology and dimensions. During the study period, Venus-P valve was the only self-expanding valve available in our center.

### Procedural Considerations

Details of the Venus-P valve and delivery system have previously been described [1]. In patients deemed suitable for Venus-P valve implant, balloon interrogation of the RVOT is performed to determine the landing zone and appropriate valve size, with simultaneous right ventricular angiography and coronary compression testing. When the intended implant location was transannular, a valve diameter of 2 to 4 mm larger than that of the balloon waist was selected. For supra-annular implant, a valve diameter equal to or up to 2 mm larger than the balloon diameter at mid main pulmonary artery (MPA) is applied. Additionally, valve sizing is influenced by other considerations, including geometry of the RVOT, expansibility in systole, stability of the sizing balloon within the RVOT, underlying diagnosis, and ensuring that the dimensions of the proximal and distal flare of the selected valve exceeds the systolic angiographic dimensions and balloon diameters at anticipated deployment site. The choice of valve length depends on the valve position and corresponding space from RVOT to MPA bifurcation based on lateral angiographic projection.

### Data Collection

Data collected included demographics, functional status, complications related to the procedure, and relevant echocardiographic and MRI parameters. Echocardiographic data included right ventricular (RV) function, severity of tricuspid regurgitation (TR), estimated right ventricular systolic pressure by TR gradient, transpulmonary valve gradient, and degree of pulmonary regurgitation (PR). Echocardiographic evaluation of PR was based on (1) jet width and length in relation to pulmonary annulus; (2) site of diastolic flow reversal in PA; (3) PR velocity waveform density and contour [12, 13]. Cardiac MRI data included indexed left ventricular (LV) and RV volumes, LV and RV ejection fraction (EF), LV to RV end-diastolic indexed volume ratio, and pulmonary regurgitant fraction (RF). Severity of RF was defined as follows: none/trivial (RF < 10%), mild (RF < 20%), moderate (RF 20–30%), and severe (RF > 30%). Cardiac output (CO) was estimated at a heart rate of 60 beats per minute. Ventricular tachycardia (VT) was defined as ≥ 4 consecutive beats at a rate ≥ 120 beats per minute and further classified as non-sustained VT (NSVT) if < 30 s or sustained VT (SVT) if ≥ 30 s or requiring termination earlier due to hemodynamic instability.

### Evaluation of Valve Sizing and Deformation Post-deployment

**Fig. 1:**
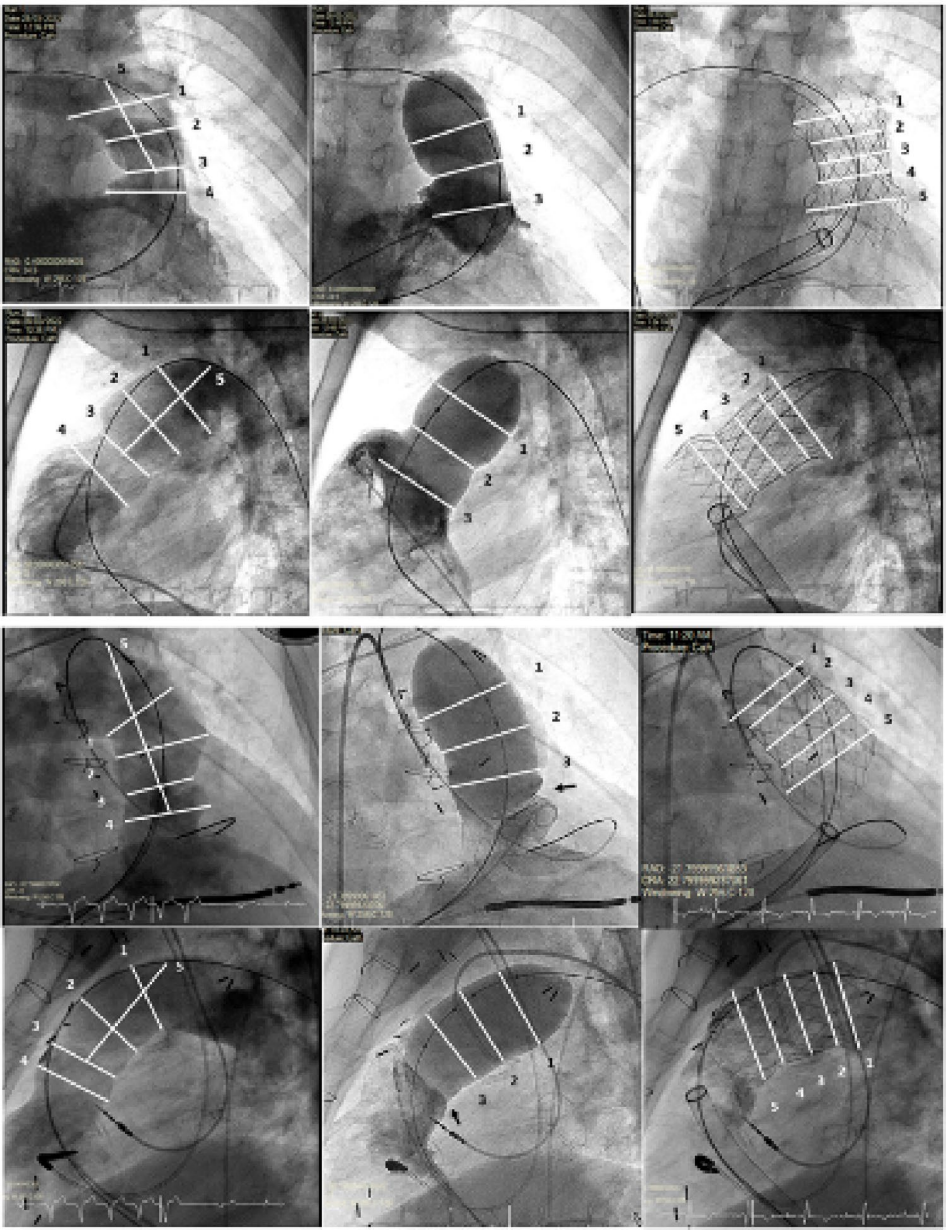
Assessment of RVOT morphology by balloon interrogation and measurement of deployed valve diameters. **a**–**f** Example of transannular implant: **a** Measurements of initial angiogram in the frontal projection at distal MPA [1], mid-MPA [2], annulus [3], 10 mm below annulus [4], and length from annulus to roof of MPA [5]. **b** Measurement of sizing balloon at 15 mm above balloon waist [1], balloon waist [2], and 15 mm below balloon waist [3]. **c** Measurements of deployed valve at outflow flare (V1), valve outflow border (V2), valve coaptation (V3), valve inflow border (V4), and inflow flare (V5). **d**–**f** Corresponding measurements in the lateral projection. **g**–**l** Example of supra-annular implant with similar measurements outlined above

The RVOT shape was classified into pyramidal, tubular, funnel, convex, or hourglass, based on pre-procedure cross-sectional assessment and/or angiographic assessment [14]. Angiographic measurements of the RVOT in systole was performed at the level of the (1) distal MPA, (2) mid-MPA, (3) pulmonary annulus, (4) 10 mm below annulus, and (5) length from annulus to distal MPA in two orthogonal planes (Fig. 1). The annular level is identified based on visualized native leaflet tissue or transition between muscular RVOT/RV myocardium and MPA. The angiographic frame used for end-systolic measurements was taken at the time of maximum expansion of the MPA or corresponding to end of T wave on ECG. Second, the balloon diameter during RVOT interrogation was measured in orthogonal planes ensuring complete occlusion of RVOT. For transannular implant, measurements were obtained at the balloon waist and 15 mm above and below the waist. For supra-annular implant, balloon diameter corresponding to mid-MPA and 15 mm above and below were measured (Fig. 1). Third, the deployed valve was measured from the distal to proximal flares in both systole and diastole at five levels (V1–V5) represented in Fig. 1.

To evaluate valve sizing, the percentage increase in selected valve diameter compared to the larger diameter of balloon waist (transannular implant) or mid-balloon diameter with complete RVOT occlusion (supra-annular implant) from two orthogonal plane was calculated (Fig. 1). The percentage of *valve ‘oversizing’* (%) was calculated as follows: 100 × ([valve diameter/balloon waist or mid balloon diameter] − 1) [15]. The *eccentricity index* (%) was calculated as 100 × (1 − [minimum valve diameter/maximum valve diameter]). The *expansion ratio* was calculated as fluoroscopy-derived waist diameter/designed valve diameter in both the AP and lateral projections [16].

To assess the impact of fluoroscopic angulation on valve diameters in the frontal plane, the 32-mm, 34-mm, and 36-mm Venus-P valves were imaged in cranial and oblique projections from 0 to 30°, maintaining a source-image distance (SID) between 90 and 94 cm. (Supplemental Table 1) Changes in angulations had minimal effect on measurements at the cylindrical straight valve section (V2–V4) with a median difference of 0 (− 0.2, 0.2) mm. There is a minor decrease in the outflow flare diameters and increase in inflow diameter at 20 to 30° cranial and/or oblique angulations with a median difference of − 0.1 (− 0.4, 0.3) mm and 0.35 (0.1, 0.7) mm, respectively (Supplemental Table 1). For these reasons, analysis of expansion ratio was primarily focused at the functional straight valve section (V2–V4) in orthogonal projections. Changes in inflow and outflow flares are mainly analyzed using measurements in the lateral projection.

**Table 1:** Baseline characteristics of total cohort (n = 42)

Age at procedure, year	39.6 (27.3, 58.6)
Weight at procedure, kg	69.8 (59.8, 85.3)
Height at procedure, cm	167 (160, 173.3)
Underlying cardiac diagnosis	
Tetralogy of Fallot or variant	33 (79)
Valvar pulmonary stenosis	7 (17)
Pulmonary atresia, intact ventricular septum	1 (2)
Carcinoid heart disease	1 (2)
*Co-morbid conditions	
None	16 (38)
**Pre-procedure arrhythmia	15 (36)
22q11 deletion	3 (7)
Diabetes	2 (5)
Renal failure	2 (5)
Obesity	3 (7)
Respiratory disease	1 (2)
Other	9 (21)
RVOT anatomic shape	
Type I (Pyramidal)	14 (33)
Type II (Tubular)	17 (40)
Type III (Funnel)	4 (9)
Type IV (Convex)	2 (4)
Type V (Hourglass)	6 (14)

Values are median (Q1–Q3) or *n*(%)

*Patients may have multiple co-morbidities

**Includes atrial and ventricular arrhythmia, implantable cardioverter defibrillator, or pacemaker implant

### Statistics

Continuous variables were expressed as mean (standard deviation) or median (interquartile range) and data distribution was checked for normality using the Shapiro–Wilk Test. Data that are normally distributed are compared using paired *t* test and data that are non-normally distributed are compared with the Wilcoxon paired test. Categorical variables were presented as frequencies and percentages and compared using the chi-squared test. The two-tailed *p* < 0.05 represented statistical significance. The relationships between the degree of valve oversizing and expansion of the valve at different levels were assessed using the Coefficient of determination, R2.

## Results

### Baseline Characteristics

Patient characteristics, NYHA functional status, and imaging parameters are presented in Tables 1 and 2. The majority of patients had repaired TOF (79%). All had severe pulmonary regurgitation as a result of prior surgical repair (*n* = 38) or transcatheter balloon dilation of stenotic PV (*n* = 2), apart from two who had no prior intervention. Three patients had mild (*n* = 1) to moderate (*n* = 2) concurrent valvar or subvalvar stenosis. In the two patients with moderate RVOT obstruction, decision for interim palliation with percutaneous PVR was due to high surgical risk and significant symptom burden. Two patients had branch PA stenosis, one with an existing RPA stent. Six patients had more than moderate tricuspid regurgitation (TR). Based on echocardiographic (*n* = 42) and MRI evaluation (*n* = 35), 25 (60%) patients had mild (*n* = 20) to moderate (*n* = 5) RV impairment and 12 (29%) patients had concurrent mild LV dysfunction. Pre-procedure MRI was not performed in 7 patients due to non-compatible ICD or pacemaker leads (*n* = 5) or availability of prior CT (*n* = 2). Thirteen patients (31%) had pre-existing documented supraventricular and/or ventricular tachycardia at baseline and all apart from one patient were on a beta-blocker.

**Table 2:** Hemodynamics and functional status pre- and post-TPVR

	Baseline, *n* = 42	6–12 weeks post, *n* = 41	6–18 months post, *n* = 27	3.0 ± 1.6 years post, *n* = 19
Echocardiographic evaluation				
RV to PA pressure gradient, mmHg	15.0 (9.4)	13.5 (6.7)	14.6 (10.0)	13.8 (8.3)
Estimated RVSP, mmHg + RAp	27.4 (11.1)	26.3 (8.7)	24.1 (6.8)	26.6 (11.3)
No pulmonary regurgitation	0	30	16	9
Mild pulmonary regurgitation	0	11	11	9
Moderate pulmonary regurgitation	0	0	0	0
Severe pulmonary regurgitation	42 (100)	0	0	0

	Baseline, *n* = 35	1.2 ± 0.5 years, *n* = 20	Pre- and post-PVR, *p* value (*n* = 20)
MRI evaluation			
LVEDVi, ml/m^2^	80.7 (20.5)	78.5 (16.3)	0.388
LVESVi, ml/m^2^	36.7 (13.1)	32.5 (9.9)	0.022
LV Stroke Volume, ml/m^2^	44.0 (9.2)	48.3 (12.9)	0.018
LV Ejection Fraction, %	56.3 (17.5) + B16:B17	59.2 (4.9)	0.022
Cardiac Index, L/min/m^2^	2.6 (0.5)	2.9 (0.8)	0.064
RVEDVi, ml/m^2^	146.0 (30.0)	107.2 (19.12)	< 0.001
RVESVi, ml/m^2^	76.1 (22.4)	57.8 (16.8)	< 0.001
RV Stroke Volume, ml/m^2^	70.3 (15.9)	49.9 (6.5)	< 0.001
RV Ejection Fraction, %	50.2 (7.5)	46.7 (7.1)	0.054
PR Regurgitation Fraction, %	42.6 (10.2)	4.3 (1.4)	< 0.001
RVEDVi:LVEDVi ratio	1.9 (0.5)	1.4 (0.3)	< 0.001

	Baseline, *n* = 42	Post-PVR, *n* = 39*
NYHA classification		
NYHA 1	21 (50)	33 (84)
NYHA 2	13 (30)	5 (12)
NYHA 3	8 (19)	1 (33)
NYHA 4	0	0

All values are expressed as mean (SD) or number (%), *p* values were obtained using paired *t* test and Wilcoxon paired test after normality test with the Shapiro–Wilk Test

*RV* right ventricular, *PA* pulmonary artery, *LV* left ventricular, *EDVi* indexed end-diastolic volume, *ESVi* indexed end-systolic volume

*Patients who died or required surgical valve replacement were excluded

### Perioperative Result

The Venus-P valve was successfully implanted in all but one patient, in whom the valve migrated proximally to an unsatisfactory position and was surgically replaced later the same day. The diameters of Venus-P valves implanted are as follows: 30 mm (*n* = 1), 32 mm (*n* = 16), 34 mm (*n* = 15), and 36 mm (*n* = 10). Valves with 30 mm length (*n* = 11) was used in the initial experience, but the 25 mm length was implanted in the majority of the cohort (*n* = 31) in later experience particularly with preference toward supra-annular deployment. A transfemoral approach was utilized in 40 (95%) procedures and a right internal jugular approach in 2(5%) procedures. Valve deployment was from LPA in 34 (81%) patients and from the RPA in 8 (19%) patients, achieving a transannular position in 33(79%) patients and supra-annular position in 9 (21%) patients. In 8 patients, the outflow flare was expanded within the proximal branch pulmonary artery in order to maintain stability in complex RVOT anatomy or to achieve supra-annular deployment with no compromise to valve function. One patient required recapture of the valve with subsequent successful deployment during the same procedure.

The median direct gradient across the valve was 3 (0.8) mmHg at baseline and 2 (0.4) mmHg post-Venus-P valve implant (*p* = 0.02). The RV:Ao ratio was 0.4 (0.3, 0.5) at baseline with no significant change observed post-valve implant (*p* = 0.22). Three patients had pre-existing moderate RVOT obstruction: one with valvar PS had complete elimination of gradient after balloon sizing/valvoplasty and two patients with subvalvar stenosis demonstrated no significant change in pre- and post-implant gradient. Post-implant MPA angiography (*n* = 38) and transoesophageal echocardiogram (*n* = 4) showed no significant valvar or paravalvar regurgitation. Predischarge echocardiogram for the 41 patients with stable valve position confirmed good valve function with no or mild pulmonary regurgitation. There were no cases of NSVT or SVT captured on in-patient telemetry or pre-discharge ECG.

### Evaluation of Valve Sizing and Valve Expansion/Deformation

In the total cohort, the geometry of the RVOT was mostly tubular (40%) or pyramidal (33%) in shape. From baseline angiography, the mean diameter at pulmonary annulus, main pulmonary artery (MPA), and distal MPA in the frontal projection was 28.8 ± 3.4 mm, 32.4 ± 4.8 mm, and 34.8 ± 7.2 mm in the frontal projection and 27.3 ± 4.1 mm, 31.5 ± 5.5 mm, and 33.4 ± 9.5 mm in the lateral projection. The average distance from annulus to PA bifurcation (*n* = 36) was 37.0 ± 10.5 mm in the lateral projection. Balloon interrogation of the RVOT showed a waist at annulus of 28.1 ± 2.9 mm in the frontal projection and 28.6 ± 2.6 mm in the lateral projection.

The extent of frame expansion and valve geometry in relation to valve oversizing were analyzed in 41 patients according to implant position. In 32 patients who received a transannular implant (*n* = 32), the difference between the balloon waist diameter and annulus diameter on initial angiography was 0.15 (− 3, 2.1) mm in the AP projection and 1.5 (− 0.2, 3.8) mm in the lateral projection. The selected valve size was a median of 4 (3.5, 6) mm larger than the maximum balloon waist measured in orthogonal projections, which translated to a median of 15 (11, 21) % valve oversizing. In the 9 patients with supra-annular implant, the median annulus diameter was 28.7 (27.9, 29.6) mm and 26.8 (23.6, 31.31) mm in the frontal and lateral planes. The mid-MPA diameter measured a median of 36.4 (35.4, 39.9) mm in the frontal plane and 32.6 (29.8, 37.2) mm and the difference between the balloon diameter and mid-MPA angiographic diameter on initial angiography was median of − 3.9 (− 5.7, − 1.2) mm in the AP projection and 1.4 (− 2.4, 3.8) mm in the lateral projection. Median valve oversizing was 0 (− 3, 4) %.

**Fig. 2:**
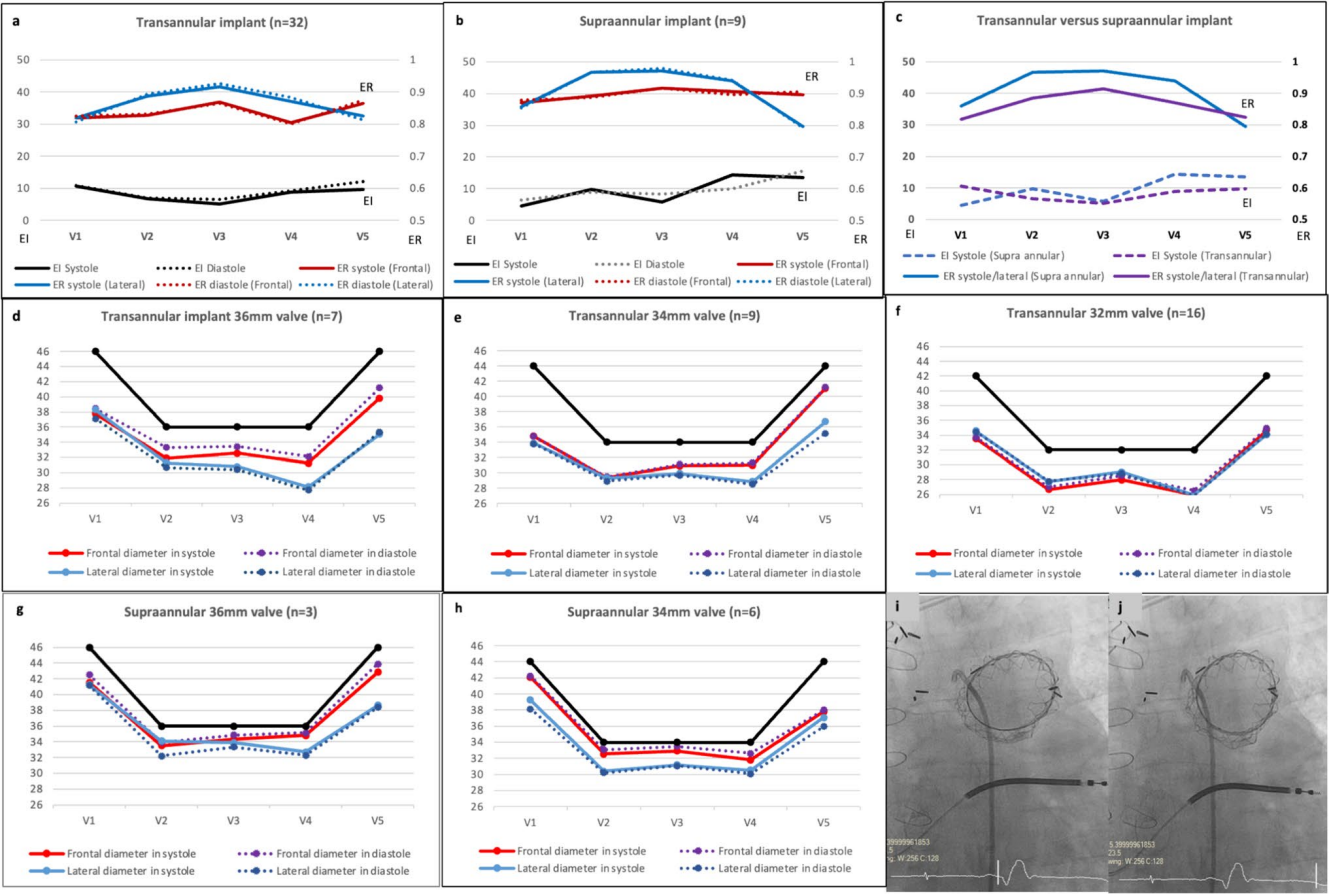
Valve expansion and geometry in relation to implant position and valve sizes. **a**–**c** Mean Expansion ratio (ER) and Eccentricity Index (EI) of deployed valve when implanted in **a** transannular and **b** supra-annular position. **c** Comparison of valve expansion and eccentricity according to implant position. **d**–**h** Mean diameters of undeployed valve (black line) versus deployed valve (colored lines) at outflow flare (V1), valve outflow (V2), valve coaptation level (V3), valve inflow border (V4), and inflow flare (V5) according to valve size and implant position in orthogonal projections during systole and diastole. **i**, **j** En Face view of a 36-mm Venus-P valve implanted in supra-annular position showing an elliptical configuration at end diastole (**i**) and more circular shape at end systole (**j**)

Overall, a larger degree of valve oversizing correlated weakly with lower expansion at the valve (V4) coaptation level. (Supplemental Fig. 1b, R2 = 0.12) The mean expansion ratio (ER) and eccentricity Index (EI) of the valve at different levels (V1–V5) are shown in Fig. 2. As expected, the valve had lowest expansion at the inflow and outflow flares. The extent of deformation at the inflow and outflow flares corresponded weakly with increased eccentricity at the same level. (Supplemental Fig. 1d and f) Deformation of both flares produces a convex configuration of the cylindrical valve section, allowing the greatest degree of expansion and circularity at the valve coaptation level (V3) in both systole and diastole (Fig. 2a–c).

When analyzed according to implant position and valve sizes, there were different patterns of frame deformation (Fig. 2d–h). Supra-annular implants showed significantly higher expansion ratio from valve inflow to outflow (V2–V4) compared to transannular implants. The valves were also more circular at coaptation level (V3) in the supra-annular position (Fig. 2c). The 32-mm valves were implanted only in the transannular position with more uniform deformation observed at all levels (V1–V5) compared to the larger 34-mm and 36-mm valves (Fig. 2d–f). The larger valves were used at transannular or supra-annular position and appeared more constrained at the proximal flare (V4–V5) with greater ellipticity in the anterior–posterior dimension compared to outflow flare (V1–V2).

There was no correlation between the eccentricity index or valve expansion ratio at valve coaptation level (V3) and direct transpulmonary valve gradient measured post-valve release or echocardiographic transvalvar gradient at last follow-up.

### Follow-Up

The duration from procedure to last clinic review was 20.3 ± 19.6 months. Two patients died during the study period. One patient with repaired Tetralogy of Fallot, ICD implant, and history of recurrent Hickman line infection died 4.5-month post-valve implant due to multi-organ failure related to severe sepsis with Candida tropicalis endocarditis. This patient was TPN dependent for nutrition with an underlying diagnosis of enteric failure. Another patient with neuroendocrine tumor of the terminal ilium with liver metastases and carcinoid heart disease died 6 months after PVR due to aggressive tumor progression. In the remaining 39 patients, an improvement in NYHA functional class was observed in 40% of the cohort and the rest reported no change in functional status (Table 2). Echocardiographic evaluation showed consistently mild or no pulmonary valve regurgitation in all patients and a reduction in the degree of TR in two of the six patients who had at least moderate TR prior to PVR. There was no progression in RVOT gradient in the two patients with pre-existing subvalvar muscular obstruction. MRI evaluation at 1.2 ± 0.5 years post-valve implant showed statistically significant improvement in RV volumes, biventricular stroke volume, and LV ejection fraction post-PVR (Table 2). None reported significant palpitations during follow-up apart from one patient who had sustained VT requiring PPM upgrade to ICD.

### Complications

There was one major (2.3%), one moderate, and 3 minor (7.1%) complications in the group. Acute complications included proximal migration of a 34 × 25 mm Venus-P valve to an unsatisfactory proximal position in a patient with short MPA and pyramidal RVOT resulting in moderate paravalvar regurgitation and frequent ventricular ectopy during recovery on the ward. We made the decision to surgically replace the valve the same day. Additionally, one patient had a small pericardial effusion unrelated to device position which was treated conservatively and another patient with biventricular dysfunction had a short period of non-invasive positive pressure ventilation. Late complications occurred in three patients: one patient with repaired Tetralogy of Fallot, moderate RV dysfunction, pre-existing atrial arrhythmias, and permanent pacemaker (PPM) had sustained ventricular tachycardia 3-week post-transannular valve implant, leading to upgrade of his PPM to an implantable cardioverter defibrillator (ICD). Another patient was treated with oral anticoagulation for thrombus formation on a single valve leaflet diagnosed at 3-month review and without functional effect on the valve. There were no interval changes in transvalvar gradient or regurgitation observed at 2-year follow-up in this patient. Frame fracture was found on fluoroscopy in one patient with calcified patch/RVOT with no compromise to valve function.

## Discussion

The use of Venus-P Valve for treatment of severely regurgitant and dysfunctional RVOT is expanding worldwide although published experience remains limited. Our outcomes are comparable to those of early series, with 98% procedural success and good valve function at medium-term follow-up. Compared to our previously published cohort of patients who underwent balloon-expandable PVR, the median age for Venus-P valve was approximately a decade older [17]. This observation may reflect a parallel change in referral patterns for TPVR in asymptomatic older patients or symptomatic patients with co-morbidities with the availability of percutaneous option for larger dysfunctional RVOT.

**Fig. 3:**
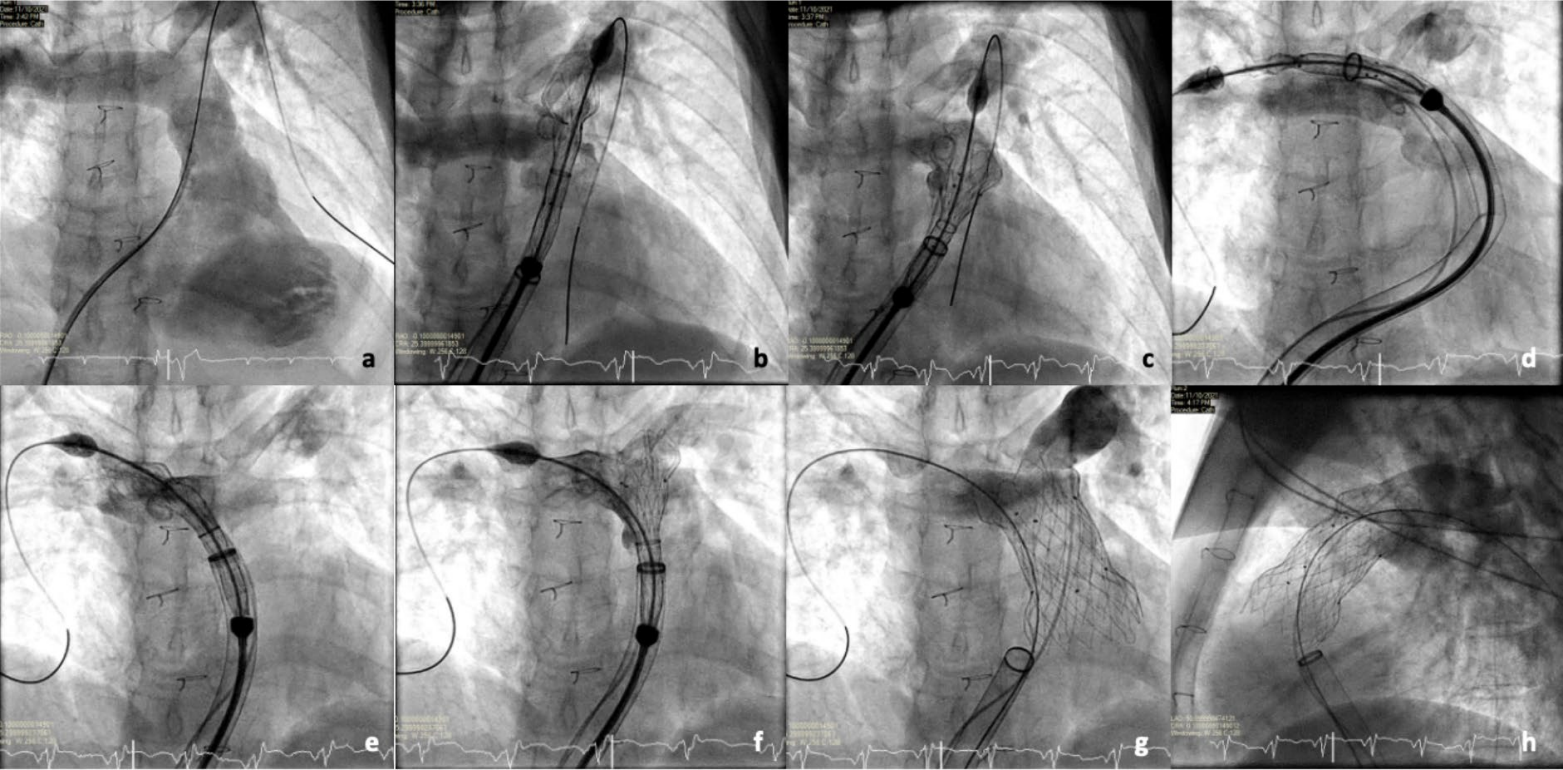
Example of pulmonary approach impacting valve deployment. **a**–**c** Initial LPA approach with unfavorable angle to RVOT resulting in proximal valve migration during expansion of distal flare. **d**–**f** Valve deployment from RPA with more coaxial alignment to RVOT allowing expansion of distal flare to blossom toward LPA/bifurcation. **g**, **h** Optimal expansion of Venus-P valve in alignment with RVOT

With increasing experience over the study period, case selection has extended to more challenging anatomies and high-risk patients. Here, we discuss procedural insights and modifications that may improve efficiency and mitigate complications. Balloon interrogation of the RVOT remains integral prior to Venus-P valve implant, allowing real-time appreciation of tissue distensibility of the outflow tract, adequacy of paravalve/balloon sealing, relationship to coronary arteries, and identification of resistant proximal or distal obstruction that may not be relieved by valve placement. While valve deployment from the left pulmonary artery was the standard approach in the early stage, our practice has evolved to base this decision on several features, including the RVOT angulation and pulmonary artery anatomy. The RPA approach was used in 8 patients in the later experience, which provided a more coaxial alignment to facilitate advancement of the valve assembly around the RVOT and subsequent valve expansion in the natural orientation of the outflow tract. In contrast, an unfavorable angulation between the RVOT and PA increases shear forces on the delivery system and the risk of valve dislocation toward the RV during deployment (Fig. 3). Similarly, we used the right internal jugular (RIJ) approach and RPA deployment in 2 patients who had gross right atrial (RA) dilation, severe RV impairment, and horizontal RVOT to facilitate a more stable and hemodynamically favorable deployment.

**Fig. 4:**
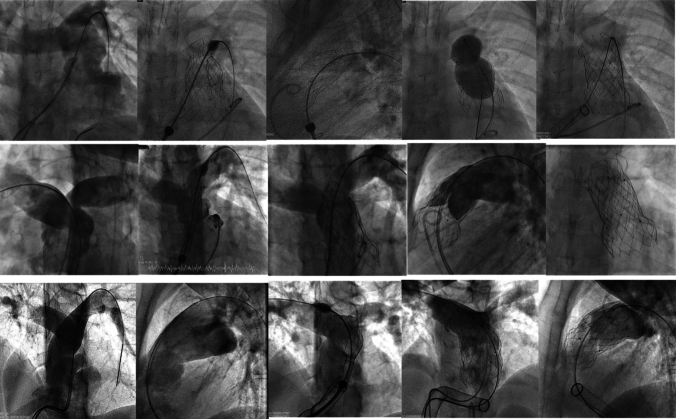
(Top panel) Example of angulated LPA with distal flare constrained in proximal LPA causing infolding of the prosthesis. The valve was recaptured with balloon and cautiously manipulated to MPA. (Middle panel) Example of prominent septal fold at PA bifurcation with distal flare constrain in proximal LPA with no compromise to valve function and spontaneous reconfiguration of distal flare in MPA observed at 6 months. (Bottom panel) Example of complex aneurysmal RVOT with superior inferior branch PA arrangement. Arrow indicates level of remnant pulmonary leaflets. Distal flare deployed in proximal RPA to avoid RV muscle encroachment with good valve function

Challenging anatomies such as short MPA, pyramidal-type RVOT, or complex PA substrate may result in deployment of the outflow flare within the proximal branch pulmonary artery. Despite constrain of the distal flare within the proximal branch PA, there was no compromise to valve function or contralateral PA flow in all apart from one case. This patient had a short convex RVOT associated with superior inferior arrangement of the branch pulmonary arteries and acute LPA angulation. Significant constrain of the distal flare within the more superior and angulated LPA resulted in infolding of the stent frame, therefore a 35 × 60 mm PTSX balloon was used to cautiously manipulate the 32 × 30 mm valve into the MPA (Fig. 4, top panel). In one case, we observed spontaneous adjustment of the distal flare and recovery to its original shape in the MPA at 6-month post-procedure (Fig. 4, middle panel). In 5 patients with supra-annular implant, the distal flare was intentionally deployed within the proximal right or left pulmonary artery with the larger diameter and most coaxial orientation to RVOT (Fig. 4, bottom panel). Complex pulmonary artery anatomy, including superior inferior arrangement, Y-shaped PA bifurcation with prominent septal fold/posterior ridge, pulmonary artery stenosis, and existing PA stent(s), may complicate valve deployment, but are no longer a deterrent for Venus-P valve implant, as shown in Supplemental Fig. 2. In high-risk patients with severely impaired RV or biventricular function and renal failure, we adopt a minimalist approach with the procedure performed under conscious sedation and minimal contrast administration [18]. In these patients, angiography was limited to check angiograms during valve deployment only (under 0.5-mls/kg contrast use) and information for valve position gained from balloon interrogation, CT/live fluoroscopy fusion, and transoesophageal echocardiogram.

Because of the dynamism of the chronically regurgitant RVOT, the implant position and degree of valve oversizing has important implications on prosthesis stability and deformation, ultimately affecting hemodynamic performance and valve longevity [7, 19, 20]. Analysis of our sizing strategy yielded several observations. Patients with transannular valve implant had significantly lower expansion at the central valve portion (V2–V4) compared to supra-annular implant likely due to a greater extent of valve oversizing applied and increased constrain at the rigid fibrous annulus or remnant leaflet evident by lower expansion at inflow (V4) compared to outflow (V2). Further, higher degree of asymmetric expansion at the inflow flare (V4–V5) with the larger 34- and 36-mm valves in the antero-posterior dimension suggest space limitation in the anterior mediastinum. In contrast, supra-annular deployment showed higher expansion (V2–V4) and stable seating of the valve despite minimal or no oversizing. Second, the degree of under-expansion or deformation at the flares does not appear to influence the expansion or eccentricity at the valve coaptation level (V3), which may allow more flexibility with valve positioning, such as constrain of outflow flare in the proximal branch PA. These findings, although exploratory and subjected to limitations of 2D cinefluoroscopic assessment, suggest that adequate deformation and apposition of one of the flared ends to vessel wall is sufficient for valve fixation, without the need for anchorage or constrain at the straight valve segment.

Whilet supra-annular valve implant appears favorable from the perspective of valve expansion and arrhythmic risk, the benefit must be carefully balanced against the risks of prosthesis instability and potential complications. Large diameter, expansile funnel-shaped RVOT, often associated with valvar PS, may necessitate transannular valve implant. Further, sufficient deformation of the inflow flare and circumferential apposition at the sinuses or proximal MPA is required to avoid paravalve regurgitation. Another observation in our limited experience with supra-annular valve implant was tilting of the covered proximal flare with prosthesis re-alignment to the MPA axis post-release, which resulted in flow turbulence at the level of the inflow. Although transvalvar gradient remained under 20 mmHg in these 3 patients, the impact of turbulent flow on leaflet kinematics and ultimately valve durability is unclear (Supplemental Video 1).

Based on our limited experience and analysis of valve configuration, we recommend valve sizing based on the balloon diameters at anticipated location of the flares and straight valve section. Emphasis is placed on adequate deformation of the flares for primary fixation, aiming for 15–20% reduction of the original flare diameter in at least one of the proximal or distal flare. This would generally correspond with a prosthesis size in the range of − 2 to + 2 mm of the balloon diameter at the waist (transannular) or mid-MPA (supra-annular deployment). The proposed 15–20% reduction in flare diameter is estimated based on the mean/median expansion ratio of proximal (V5) and distal flare (V1) in our cohort with secure valve fixation, supported by recommendations from initial bench testing of self-expanding valves in experimental RVOT models [21]. This approach exposes the straight valve section to minimal constrain thereby optimizing leaflet kinematics and durability.

## Limitations

This study has several key limitations. This is an observational, retrospective single-center review of a small cohort of patients which is subjected to biases associated with patient selection and unequal sample sizes in subgroup analyses. Additionally, evolving anatomic selection criteria and refinement in procedural techniques over the course of the eight-year study period may influence patient/valve outcomes. Second, analysis of the deployed valve geometry and expansion is subjected to limitations of 2D fluoroscopic views which are factored into consideration in methodology and analysis. Computed tomography does not form part of our routine monitoring due to the radiation risks, although the 3D dataset would provide more accurate dimensions of the deployed prosthesis. Third, there may be changes in valve configuration or expansion over time, which was not explored in this study. Multicenter experiences on technical implant details and longer-term follow-up are required to identify best practices for achieving long-term valve durability.

## Conclusion

Our experience demonstrates broad applicability of the Venus-P valve in different RVOT and pulmonary artery anatomies with good mid-term outcomes, albeit with variation in final prosthesis position and deformation. Analysis of the deployed configuration showed that the prosthesis can be anchored safely by adequate deformation of the flared portion with minimal or no constrain at the functional valve section. Approach to Venus-P valve sizing should place more emphasis on the degree of deformation at the inflow or outflow flares at the intended deployment location for anchorage, instead of oversizing based solely on annular or balloon waist diameter. This may allow maximal and circular expansion of the valve section to facilitate optimal leaflet kinematics and hemodynamics. The effect of different implantation position and prosthesis geometry on longer-term hemodynamic profile and relevant patient outcomes requires further evaluation to inform optimal implantation strategies.

## Supplementary Information

Below is the link to the electronic supplementary material.

Supplementary file1 (DOCX 1283 KB)

Supplementary file2 (PPTX 6942 KB)

## Data Availability

No datasets were generated or analysed during the current study.

## References

[1] Morgan G, Prachasilchai P, Promphan W, Rosenthal E, Sivakumar K, Kappanayil M, Sakidjan I, Walsh KP, Kenny D, Thomson J, Koneti NR, Awasthy N, Thanopoulos B, Roymanee S, Qureshi S (2019) Medium-term results of percutaneous pulmonary valve implantation using the Venus P-valve: international experience. EuroIntervention 14:1363–1370 30248020 10.4244/EIJ-D-18-00299

[2] Goldstein BH, McElhinney DB, Gillespie MJ, Aboulhosn JA, Levi DS, Morray BH, Cabalka AK, Love BA, Zampi JD, Balzer DT, Law MA, Schiff MD, Hoskoppal A, Qureshi AM (2024) Early outcomes from a multicenter transcatheter self-expanding pulmonary valve replacement registry. J Am Coll Cardiol 83:1310–1321 38569760 10.1016/j.jacc.2024.02.010

[3] Martin C, Sun W (2017) Transcatheter valve underexpansion limits leaflet durability: implications for valve-in-valve procedures. Ann Biomed Eng 45:394–404 27734178 10.1007/s10439-016-1738-8 PMC5300953

[4] Fukui M, Bapat VN, Garcia S, Dworak MW, Hashimoto G, Sato H, Gossl M, Enriquez-Sarano M, Lesser JR, Cavalcante JL, Sorajja P (2022) Deformation of transcatheter aortic valve prostheses: implications for hypoattenuating leaflet thickening and clinical outcomes. Circulation 146:480–493 35862182 10.1161/CIRCULATIONAHA.121.058339

[5] Azadani AN, Reardon M, Simonato M, Aldea G, Nickenig G, Kornowski R, Dvir D (2017) Effect of transcatheter aortic valve size and position on valve-in-valve hemodynamics: an in vitro study. J Thorac Cardiovasc Surg 153(1303–1315):e1301 10.1016/j.jtcvs.2016.12.057 28283233

[6] Garcia S, Fukui M, Dworak MW, Okeson BK, Garberich R, Hashimoto G, Sato H, Cavalcante JL, Bapat VN, Lesser J, Cheng V, Newell MC, Goessl M, Elmariah S, Bradley SM, Sorajja P (2022) Clinical impact of hypoattenuating leaflet thickening after transcatheter aortic valve replacement. Circ Cardiovasc Interv 15:e011480 35236097 10.1161/CIRCINTERVENTIONS.121.011480

[7] Fukui M, Cavalcante JL, Bapat VN (2024) Deformation in transcatheter heart valves: clinical implications and considerations. J Cardiol 83:351–358 38432474 10.1016/j.jjcc.2024.02.011

[8] Egbe AC, Pislaru SV, Pellikka PA, Poterucha JT, Schaff HV, Maleszewski JJ, Connolly HM (2015) Bioprosthetic valve thrombosis versus structural failure: clinical and echocardiographic predictors. J Am Coll Cardiol 66:2285–2294 26610876 10.1016/j.jacc.2015.09.022

[9] Schievano S, Capelli C, Young C, Lurz P, Nordmeyer J, Owens C, Bonhoeffer P, Taylor AM (2011) Four-dimensional computed tomography: a method of assessing right ventricular outflow tract and pulmonary artery deformations throughout the cardiac cycle. Eur Radiol 21:36–45 20680286 10.1007/s00330-010-1913-5

[10] Alkhouli M, Hijazi ZM, Holmes DR Jr, Rihal CS, Wiegers SE (2018) Intracardiac echocardiography in structural heart disease interventions. JACC Cardiovasc Interv 11:2133–2147 30409271 10.1016/j.jcin.2018.06.056

[11] Zhou D, Pan W, Jilaihawi H, Zhang G, Feng Y, Pan X, Liu J, Yu S, Cao Q, Ge J (2019) A self-expanding percutaneous valve for patients with pulmonary regurgitation and an enlarged native right ventricular outflow tract: one-year results. EuroIntervention 14:1371–1377 30398963 10.4244/EIJ-D-18-00715

[12] Zoghbi WA, Adams D, Bonow RO, Enriquez-Sarano M, Foster E, Grayburn PA, Hahn RT, Han Y, Hung J, Lang RM, Little SH, Shah DJ, Shernan S, Thavendiranathan P, Thomas JD, Weissman NJ (2017) Recommendations for noninvasive evaluation of native valvular regurgitation: a report from the American society of echocardiography developed in collaboration with the society for cardiovascular magnetic resonance. J Am Soc Echocardiogr 30:303–371 28314623 10.1016/j.echo.2017.01.007

[13] Zoghbi WA, Asch FM, Bruce C, Gillam LD, Grayburn PA, Hahn RT, Inglessis I, Islam AM, Lerakis S, Little SH, Siegel RJ, Skubas N, Slesnick TC, Stewart WJ, Thavendiranathan P, Weissman NJ, Yasukochi S, Zimmerman KG (2019) Guidelines for the evaluation of valvular regurgitation after percutaneous valve repair or replacement: a report from the American society of echocardiography developed in collaboration with the society for cardiovascular angiography and interventions, Japanese society of echocardiography, and society for cardiovascular magnetic resonance. J Am Soc Echocardiogr 32:431–475 30797660 10.1016/j.echo.2019.01.003

[14] Schievano S, Coats L, Migliavacca F, Norman W, Frigiola A, Deanfield J, Bonhoeffer P, Taylor AM (2007) Variations in right ventricular outflow tract morphology following repair of congenital heart disease: implications for percutaneous pulmonary valve implantation. J Cardiovasc Magn Reson 9:687–695 17578725 10.1080/10976640601187596

[15] Athappan G, Patvardhan E, Tuzcu EM, Svensson LG, Lemos PA, Fraccaro C, Tarantini G, Sinning JM, Nickenig G, Capodanno D, Tamburino C, Latib A, Colombo A, Kapadia SR (2013) Incidence, predictors, and outcomes of aortic regurgitation after transcatheter aortic valve replacement: meta-analysis and systematic review of literature. J Am Coll Cardiol 61:1585–1595 23500308 10.1016/j.jacc.2013.01.047

[16] Kawamori H, Yoon SH, Chakravarty T, Maeno Y, Kashif M, Israr S, Abramowitz Y, Mangat G, Miyasaka M, Rami T, Kazuno Y, Takahashi N, Jilaihawi H, Nakamura M, Cheng W, Friedman J, Berman D, Sharma R, Makkar RR (2018) Computed tomography characteristics of the aortic valve and the geometry of SAPIEN 3 transcatheter heart valve in patients with bicuspid aortic valve disease. Eur Heart J Cardiovasc Imaging 19:1408–1418 29315371 10.1093/ehjci/jex333

[17] Hribernik I, Thomson J, Ho A, English K, Van Doorn C, Jaber O, Bentham J (2022) Comparative analysis of surgical and percutaneous pulmonary valve implants over a 20-year period. Eur J Cardiothorac Surg 61:572–579 34406369 10.1093/ejcts/ezab368

[18] Butala NM, Chung M, Secemsky EA, Manandhar P, Marquis-Gravel G, Kosinski AS, Vemulapalli S, Yeh RW, Cohen DJ (2020) Conscious sedation versus general anesthesia for transcatheter aortic valve replacement: variation in practice and outcomes. JACC Cardiovasc Interv 13:1277–1287 32499018 10.1016/j.jcin.2020.03.008 PMC7650030

[19] Groves EM, Falahatpisheh A, Su JL, Kheradvar A (2014) The effects of positioning of transcatheter aortic valves on fluid dynamics of the aortic root. ASAIO J 60:545–552 25010918 10.1097/MAT.0000000000000107 PMC4334568

[20] Pietrasanta L, Zheng S, De Marinis D, Hasler D, Obrist D (2021) Characterization of turbulent flow behind a transcatheter aortic valve in different implantation positions. Front Cardiovasc Med 8:804565 35097022 10.3389/fcvm.2021.804565 PMC8794584

[21] Biglino G, Capelli C, Binazzi A, Reggiani R, Cosentino D, Migliavacca F, Bonhoeffer P, Taylor AM, Schievano S (2012) Virtual and real bench testing of a new percutaneous valve device: a case study. EuroIntervention 8:120–128 22580256 10.4244/EIJV8I1A19

