# Supplementary material for: Single-Center Experience with Venus-P Self-expanding Pulmonary Valve: Insights on Valve Sizing and Procedural Techniques
Source: Pediatr Cardiol. 2025 Apr 2;47(2):784–94. doi: 10.1007/s00246-025-03841-5 (PMC12855437; doi:10.1007/s00246-025-03841-5)
Supplement: Supplementary file 1 — Supplementary file1 (DOCX 1283 KB) [file 246_2025_3841_MOESM1_ESM.docx]

### Title

Sizing considerations with Venus-P self-expanding pulmonary valve to enhance leaflet kinematics and haemodynamics: Outcomes and procedural insights from a single centre.

### Supplementary Information

**Supplemental Table 1** Measurements of undeployed valve in different fluoroscopic angulations.


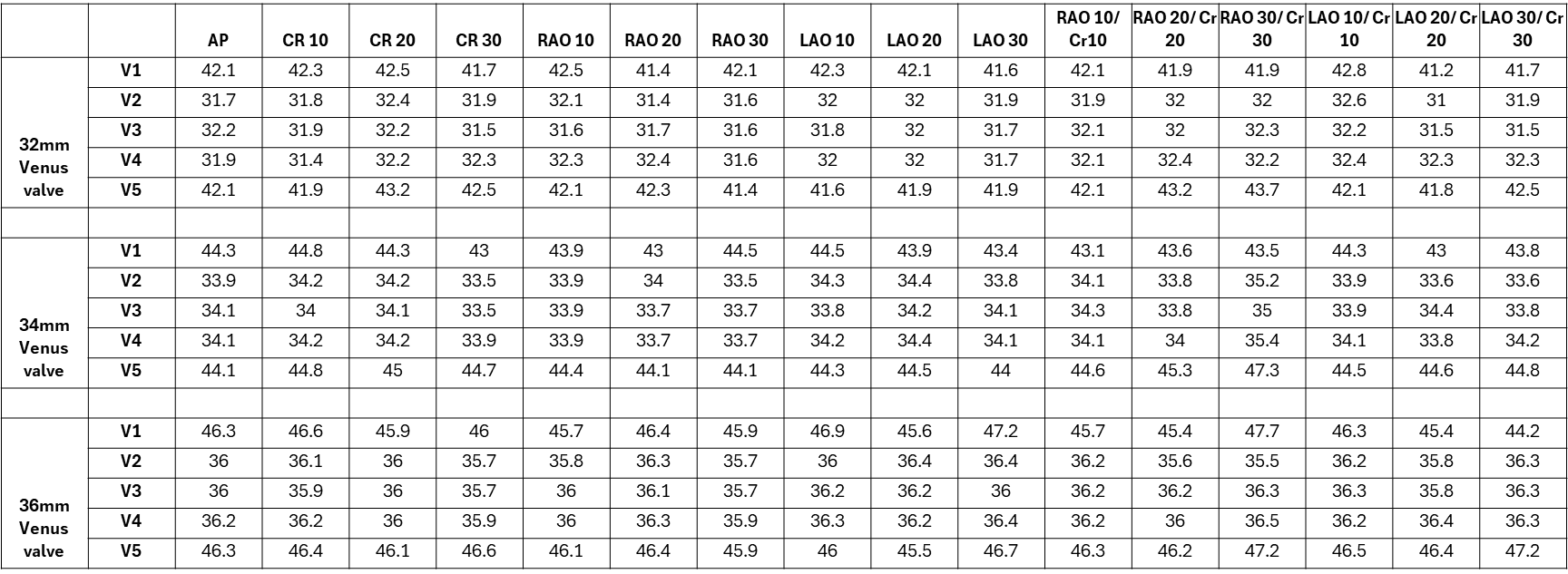


**Supplemental Figure 1** Analysis of valve oversizing versus expansion at outflow flare (a), valve coaptation (b) and inflow flare(c). Systolic expansion ratio versus valve eccentricity at outflow flare (d), valve coaptation (e) and inflow flare (f). Analysis of the degree of deformation at inflow and ouflow flares on valve expansion and geometry at coaptation level (g-j), valve outflow border (k) and valve inflow border (l).


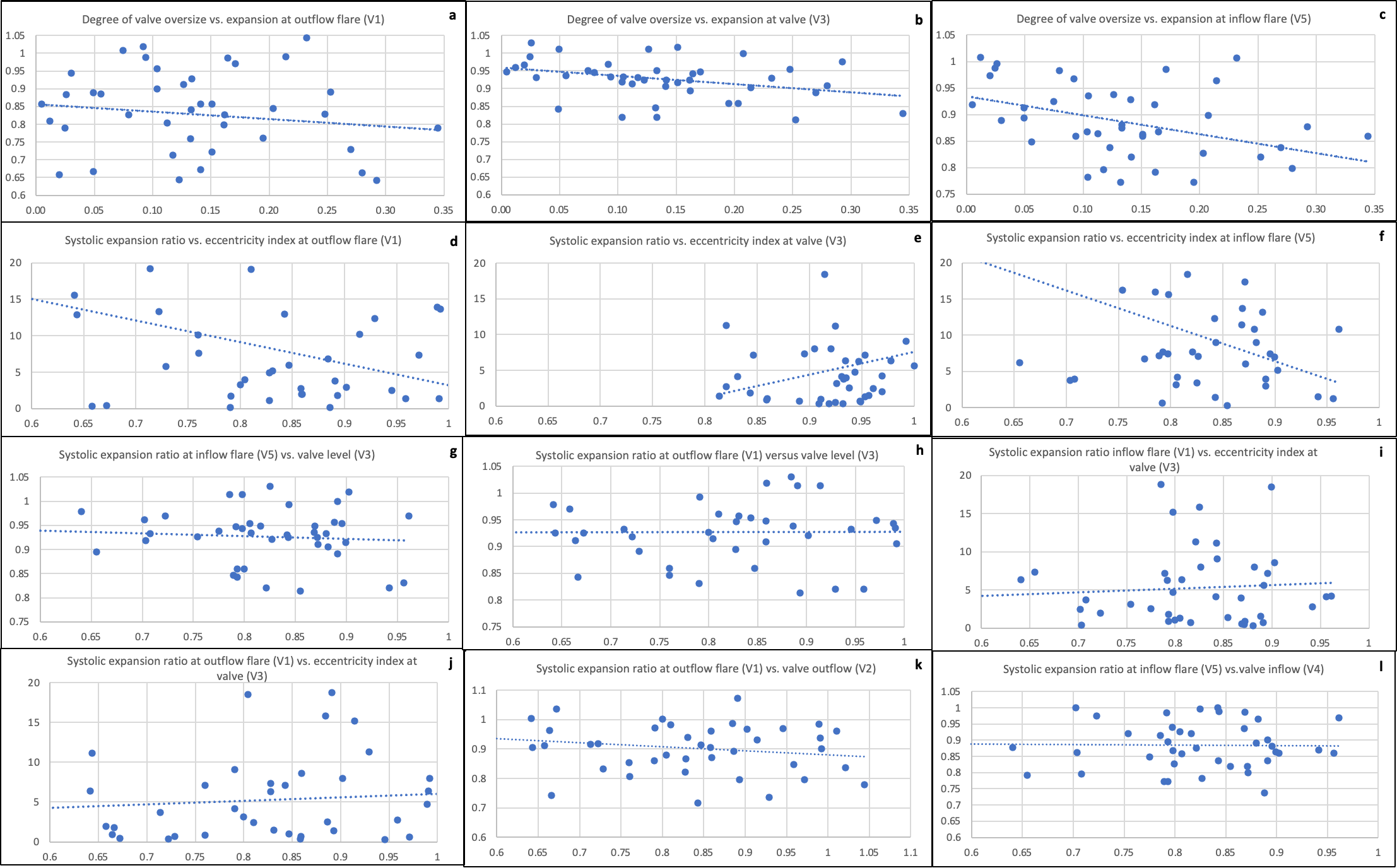


**Supplemental Figure 2** Examples of Venus-P valve implant in complex pulmonary artery substrate. (Top panel) Valve deployment from the LPA in a patient with previous RPA stent with sufficient distal MPA space for outflow flare opening. (Bottom panel) Valve deployment from distal MPA in a patient with significantly angulated LPA and mild ostial stenosis.


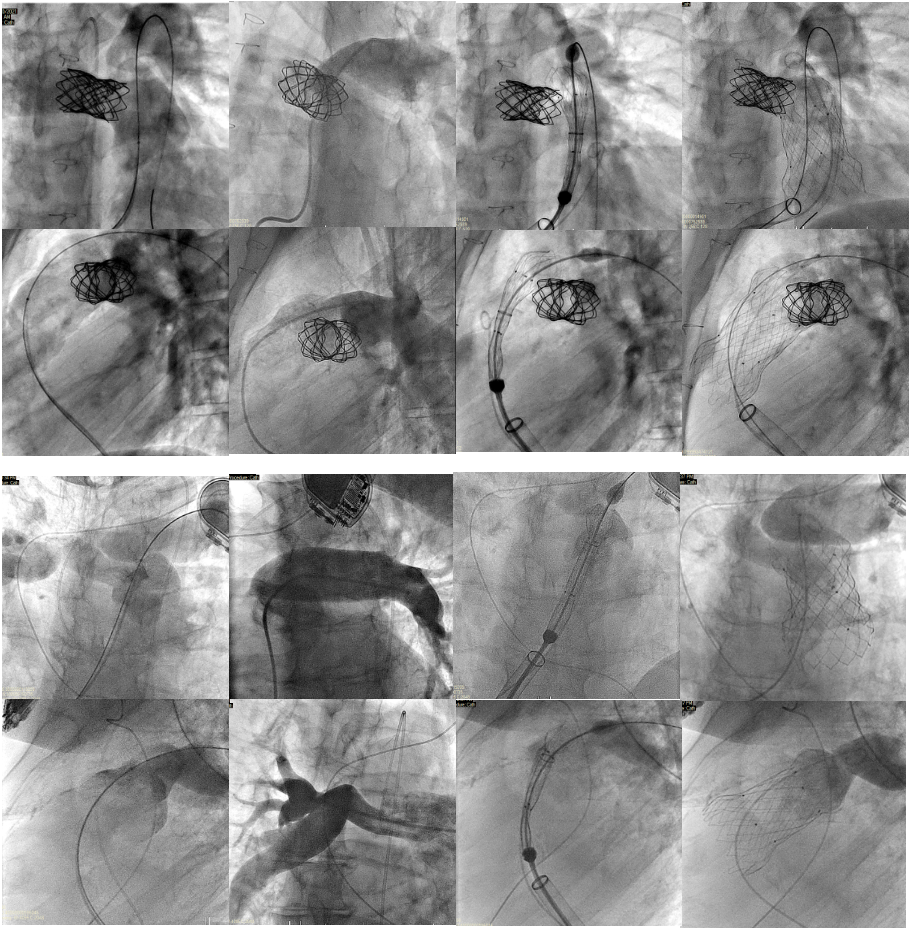


**Supplemental video 1** Anterior tilting of the valve frame in supraannular position with flow turbulence at the covered inflow flare.
