# Supplementary figures and images for: Single-Center Experience with Venus-P Self-expanding Pulmonary Valve: Insights on Valve Sizing and Procedural Techniques
Source: Pediatr Cardiol. 2025 Apr 2;47(2):784–94. doi: 10.1007/s00246-025-03841-5 (PMC12855437; doi:10.1007/s00246-025-03841-5)

## Slide 1
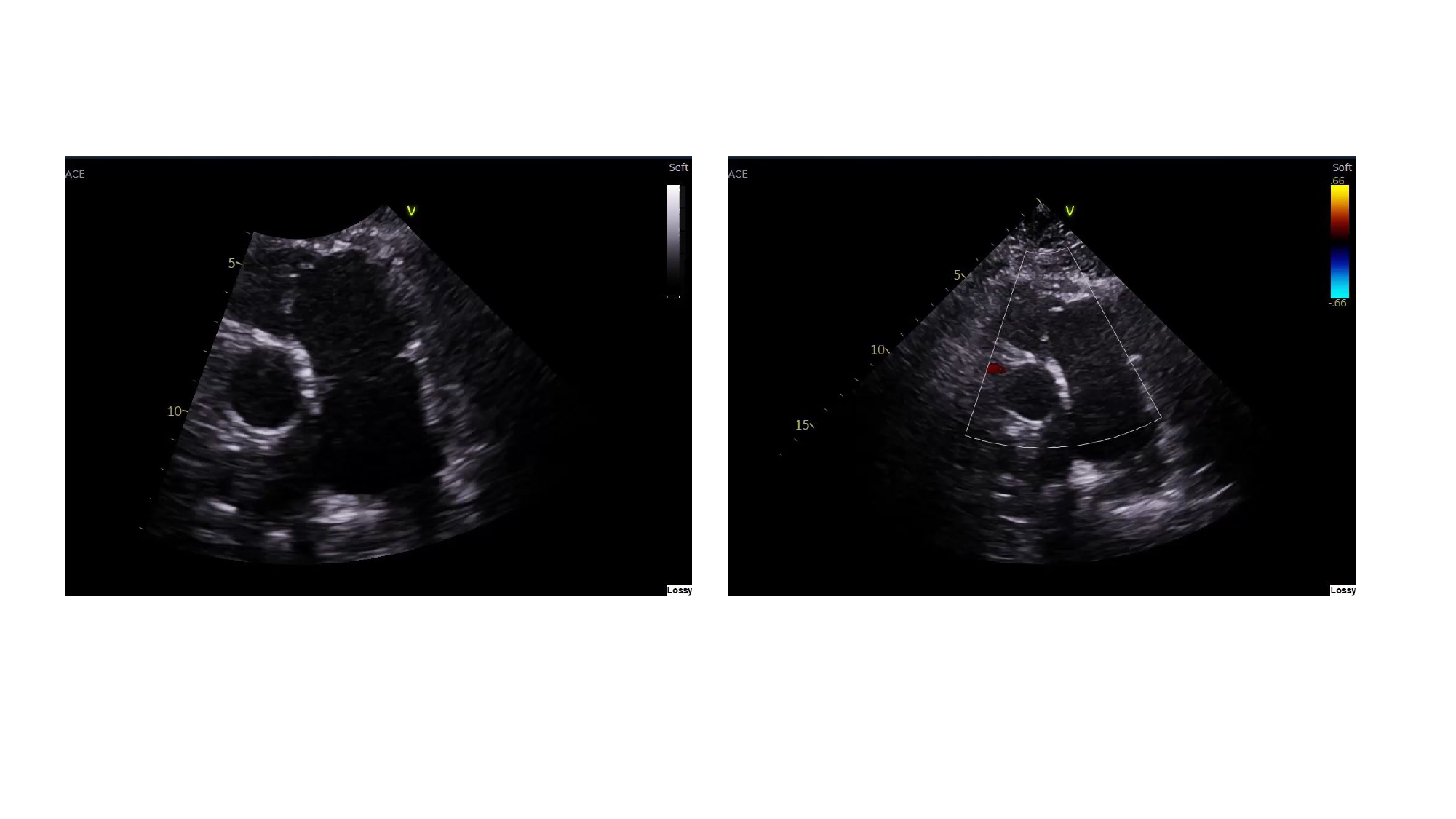

Supplement: Supplementary file 2 — Supplementary file2 (PPTX 6942 KB) [file 246_2025_3841_MOESM2_ESM.pptx]
